# New Appliances and Things Medical

**Published:** 1903-10-03

**Authors:** 


### NEW APPLIANCES AND THINGS MEDICAL.

**[We shall be glad to receive at our Office, 28 & 29 Southampton Street, Strand, London, W.C., from the manufacturers, specimens of all new preparations and appliances which may be brought out from time to time.]**

### ROSBACH WATER.

(ROSBACH COMPANY, LIMITED, 13 REGENT STREET, LONDON, S.W.)

WE have again had an opportunity of testing the good qualities of this excellent table water, and find that it still merits the high opinion we had previously formed of it. It is a natural sparkling water, bottled at the springs near Homburg, and it has all the characteristics which would induce us to highly recommend its use whenever a table water may be required. It is pure, full of sparkle, pleasant to the palate, and quite free from all objectionable qualities.

### PEARSON'S ANTISEPTIC.

(PEARSON'S ANTISEPTIC COMPANY, LIMITED, 254A HIGH HOLBORN, LONDON, W.C.)

SINCE our last notice of this valuable creolin preparation Dr. A. B. Griffiths's report on it has come to hand, showing most clearly what a potent and useful germicide it is. Both its powerful antiseptic properties and its rapidity of action are well demonstrated in the results experimentally obtained by this analyst. With a 20 per cent. solution the microbes of enteric fever, cholera, and influenza were destroyed in 2, 10, and 14 seconds respectively. With a 1 per cent. solution 60, 60, and 65 seconds respectively was sufficient time to destroy these microbes; while even when diluted to the strength of 1 in 5,000 it proved to be antiseptic. In another series of experiments a 10 per cent. solution of Pearson's Antiseptic was sprayed into the air of ordinary rooms, the number of microbes in 15 litres of this air having previously been ascertained by Hesse's well-known method to be about 300. After 10 minutes' spraying this number was reduced to 45, while after 20 minutes no microbes were discoverable. Dr. Griffiths also shows how easily this preparation destroys the poisonous ptomaines indirectly produced by microbes or bacteria.

### FERROLEUM.

(THE FERROLEUM COMPANY, LIMITED, 86 CLERKENWELL ROAD, LONDON, E.C.)

SINCE this preparation was first placed on the market twelve months ago, there has been plenty of opportunity to clinically test its value, and we are assured from inquiry that its sphere of usefulness has been wide and its efficacy undoubted. Its formula consists of cod-liver oil, phosphorus and phosphate of iron dissolved in glycerine and water; these ingredients being skilfully combined in the form of a fine emulsion. Such a combination when found to be comparatively palatable and easily assimilated, should rightly lead to its wide adoption as a reliable remedy in cases where the system requires building up. Therefore in tuberculosis, convalescence after acute disease and in many debilitated conditions "Ferroleum" proves to be of great value.

